# Optimal cutoff values of alanine aminotransferase and its derived markers for pediatric MASLD

**DOI:** 10.3389/fendo.2026.1808797

**Published:** 2026-07-22

**Authors:** Joon Young Kim, Eunju Lee, Hye Sun Lee, Young Hoon Youn, Su Jung Baik, Hyun Joo Shin, Yu-Jin Kwon, Eun Byoul Lee, Hyun Wook Chae, Kyungchul Song

**Affiliations:** 1 Gangnam Severance Hospital, Department of Pediatrics, Yonsei University College of Medicine, Seoul, Republic of Korea; 2 Biostatistics Collaboration Unit, Yonsei University College of Medicine, Seoul, Republic of Korea; 3 Gangnam Severance Hospital, Health Promotion Center, Healthcare Research Team, Yonsei University College of Medicine, Seoul, Republic of Korea; 4 Yongin Severance Hospital, Research Institute of Radiological Science and Center for Clinical Imaging Data Science, Department of Radiology, Yonsei University College of Medicine, Yongin-si, Republic of Korea; 5 Yongin Severance Hospital, Department of Family Medicine, Yonsei University College of Medicine, Yongin-si, Republic of Korea; 6 Yongin Severance Hospital, Department of Pediatrics, Yonsei University College of Medicine, Yongin-si, Republic of Korea

**Keywords:** adolescent, biomarkers, child, early diagnosis, fatty liver, liver function tests

## Abstract

**Background:**

The early detection of metabolic dysfunction-associated steatotic liver disease (MASLD) is essential. This study aims to evaluate the diagnostic utility and optimal cutoffs of alanine aminotransferase (ALT) and its derived markers, including the ALT/aspartate aminotransferase (AST) and ALT/high-density lipoprotein cholesterol (HDL) ratios, for predicting pediatric MASLD.

**Methods:**

We analyzed data from 1,158 adolescents in the National Health and Nutritional Examination Survey (NHANES) and 263 adolescents from a real-world clinical cohort. MASLD was assessed using vibration-controlled transient elastography in the NHANES cohort and abdominal ultrasonography in the clinical cohort. Logistic regression and receiver operating characteristic (ROC) curve analyses were conducted to evaluate associations and predictive performance.

**Results:**

ALT, ALT/AST, and ALT/HDL were significantly associated with MASLD in both datasets, even after adjusting for age and sex (all p < 0.001). In the NHANES cohort, the areas under the ROC curves (AUCs) for ALT, ALT/AST, and ALT/HDL were 0.73, 0.77, and 0.76, respectively, with optimal cutoff values of >14.50 for ALT, >0.74 for ALT/AST, and >0.29 for ALT/HDL in the total population. In the real-world clinical cohort, the corresponding AUCs were 0.84, 0.84, and 0.86, respectively. ALT/HDL demonstrated the highest AUC in both datasets, reaching 0.92 in females in the clinical cohort.

**Conclusions:**

ALT and ALT-derived markers are valuable for predicting pediatric MASLD. Among them, the ALT/HDL ratio demonstrated the best predictive performance, indicating that composite indices integrating markers of hepatic injury and metabolic status may enhance early detection of MASLD in children and adolescents.

## 1 Introduction

Metabolic dysfunction-associated steatotic liver disease (MASLD) is a chronic liver disease characterized by hepatic steatosis in individuals with at least one cardiovascular risk factor, excluding other causes (1). This status may be followed by hepatic inflammation, which contributes to fibrogenesis and potentially progresses to liver cirrhosis and hepatocellular carcinoma (2, 3). Furthermore, MASLD is a representative metabolic disorder driven by insulin resistance, which may exacerbate hepatic lipid accumulation, thereby impairing hepatocyte’s ability to manage metabolic stress (4). Thus, MASLD is closely associated with type 2 diabetes and cardiovascular diseases (3, 5). The prevalence of MASLD is 32.5% among the general population in the United States (US) and 20.4% among US youth (6–8). Moreover, Le et al. reported that, using linear regression to project the prevalence of MASLD by age group, 41.4% of the US population is predicted to have MASLD by 2050 (9).

Because MASLD is a progressive condition, early detection is crucial (10). Current guidelines suggest biopsy or imaging as tools to assess hepatic steatosis (1). However, a liver biopsy is invasive, particularly in children, and imaging procedures may be costly and less accessible in many global healthcare settings. Thus, the identification of relatively noninvasive and easily implemented biomarkers that can be used across diverse global healthcare settings remain important. In real-world clinical practice, simple and easily accessible laboratory biomarkers, such as aspartate aminotransferase (AST) and alanine aminotransferase (ALT), are commonly used as screening tools for liver diseases and for assessing abnormalities in liver function (11, 12).

ALT specifically reflects hepatocellular injury, including liver steatosis, and has been widely used to assess chronic liver disease (12, 13). In pediatric nonalcoholic fatty liver disease (NAFLD) guidelines, ALT has been proposed as a useful screening tool (12, 14). A cross-sectional study reported that ALT and ultrasonography have comparable diagnostic accuracy for detecting NAFLD in children with obesity (15). However, Mollstone et al. reported that significant NAFLD and fibrosis occurred even in children with normal ALT levels (16). Moreover, investigations on the diagnostic accuracy of ALT for MASLD in youth are limited. Although combined indices, such as the hepatic steatosis index (HSI) and triglyceride-to-high-density lipoprotein cholesterol ratio (TG/HDL), have also been suggested as non-invasive screening tools for NAFLD, their broader clinical utility remains constrained by the requirement for fasting laboratory measurements or the complexity of their constituent formulas, limiting their practicability in general pediatric practice (17, 18). Moreover, the ALT cutoffs previously used in children and adolescents were not optimal thresholds for hepatic steatosis, but rather values established based on the population-defined upper limit of normal (ULN). Notably, the usefulness and appropriateness of the current cutoff value have been questioned under the new concept of MASLD in children, particularly because of its limited capacity to reflect the metabolic aspects emphasized in MASLD (14, 19–21).

Meanwhile, certain trials have attempted to identify adequate indices for predicting NAFLD, proposing novel alternative indices derived from existing biomarkers in the adult population (22–24). Long et al. suggested that the ALT/AST ratio has a stronger association with the degree of hepatic steatosis observed on liver biopsy than ALT alone (25). Accordingly, the ALT/AST ratio has been suggested as a marker of NAFLD risk and severity in adults (24). In addition, Qiu et al. reported that the ALT/high-density lipoprotein cholesterol (HDL) ratio is an important predictive factor of NAFLD in adults (23). However, investigations on the predictability and optimal cutoffs of these markers for pediatric MASLD remain limited (26).

Therefore, this study aims to evaluate the efficacy of ALT-derived parameters, including ALT, ALT/AST, and ALT/HDL ratios, in predicting the diagnosis of pediatric MASLD using data from the National Health and Nutritional Examination Survey (NHANES) and real-world clinical data. We designed this study to 1) assess and compare the predictability of ALT and its derived markers with that of preexisting combined parameters, including TG/HDL and HSI, for pediatric MASLD; 2) determine optimal cutoff values for ALT and its derived markers using population-based data; and 3) apply them to real-world clinical data.

## 2 Materials and methods

### 2.1 Ethics statement

This study adhered to the ethical principles outlined in the 1975 Declaration of Helsinki and was approved by the Institutional Review Board of Gangnam Severance Hospital (GSH; IRB No: 3-2024-0368). Written informed consent was obtained from all participants in the NHANES and Gangnam Severance Hospital (GSH) cohorts. The requirement for informed consent was waived for participants from Yongin Severance Hospital (YSH) owing to the retrospective nature of the study.

### 2.2 Study participants

**Figure 1:**
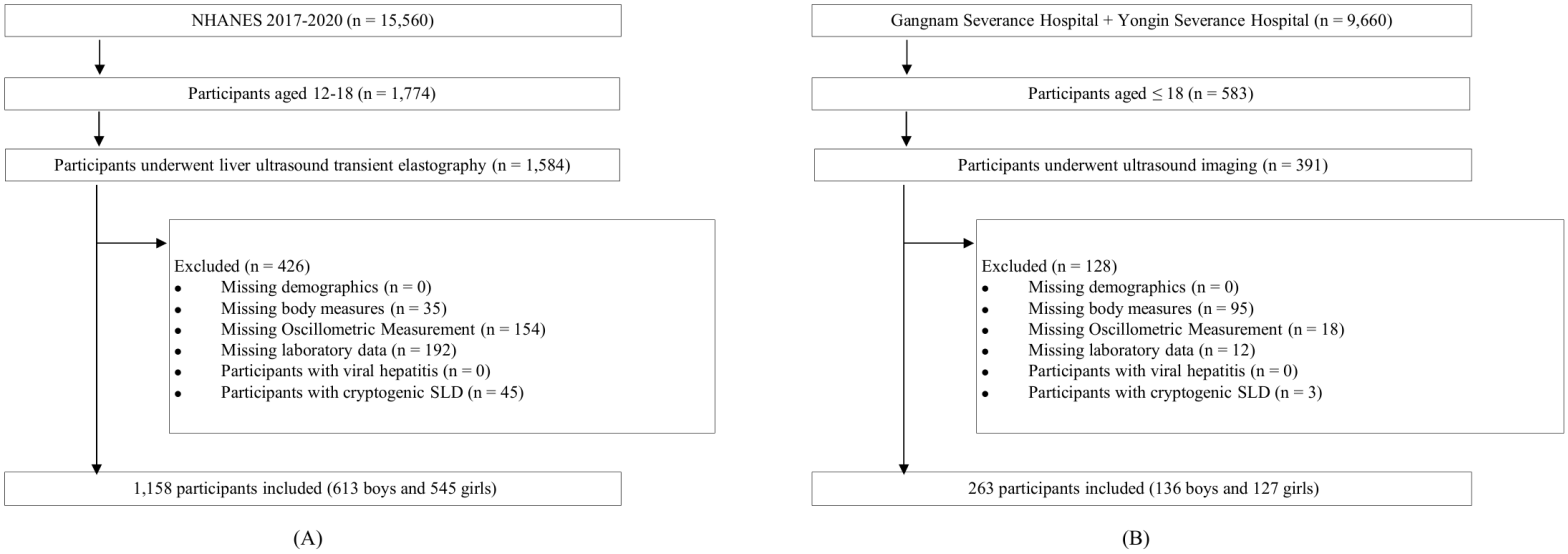
Flowchart of participants and dataset construction. **(A)** NHANES dataset. **(B)** Real-world clinic dataset. NHANES, National Health and Nutrition Examination Survey; GSH, Gangnam Severance Hospital; YSH, Yongin Severance Hospital; SLD, Steatotic liver disease.

This retrospective, cross-sectional study included two distinct cohorts. A total of 1,158 youth aged 12–18 years who participated in NHANES between 2017 and 2020 were enrolled (**Figure 1**). Additionally, 263 children and adolescents aged 18 years or younger who visited the Health Promotion Center at GSH between January 2007 and December 2023, or at YSH between September 2022 and February 2024, were included in the real-world clinical dataset.

### 2.3 Study variables

Height, weight, and body mass index (BMI) standard deviation scores (SDS) in the NHANES dataset were calculated using the Centers for Disease Control and Prevention growth reference data (27). Korean population-based reference standards were used to calculate values for the GSH and YSH datasets (28). BMI was classified into three categories: normal (<85th percentile), overweight (85th–95th percentile), and obesity (>95th percentile) (27, 28). Central obesity was defined as a waist circumference (WC) exceeding the 95th percentile for age and sex (29, 30).

### 2.4 Diagnosis of MASLD

MASLD was defined as the presence of hepatic steatosis accompanied by at least one of the following five metabolic risk factors: (A) BMI ≥ 85th percentile for age and sex, or WC ≥ 95th percentile for age and sex; (B) fasting plasma glucose ≥ 100 mg/dL, or glycated hemoglobin ≥ 5.7%; (C) elevated blood pressure (BP), defined as ≥ 95th percentile for age, sex, and height, or ≥ 130/80 mmHg in children <13 years of age and ≥ 130/85 mmHg in those ≥13 years; (D) triglycerides (TG) ≥ 150 mg/dL; and/or (E) HDL ≤ 40 mg/dL (1). Hepatic steatosis was assessed using vibration-controlled transient elastography with a FibroScan 502 V2 Touch (Echosens, Paris, France), equipped with both medium and extra-large probes for use in the NHANES cohort. Hepatic steatosis was diagnosed when the median controlled attenuation parameter (CAP) exceeded 248 dB/m (31–33). CAP values indicate the amount of liver fat, with higher values indicating greater fat accumulation. Hepatic steatosis was detected in both GSH and YSH using abdominal sonography to assess liver echogenicity. A 3.5 MHz transducer (HDI 5000, Philips, Bothell, WA, USA) was used in GSH, while a C1–8 MHz transducer (Aplio i800, Canon Medical Systems, Otawara, Japan) and a C1–6 MHz transducer (LOGIQ E10, GE Healthcare, Wauwatosa, WI, USA) were alternately employed in YSH. All radiologists adhered to a standardized protocol for the diagnosis of steatotic liver disease (SLD). Hepatic steatosis was assessed using ultrasonographic findings, including increased hepatic echogenicity relative to the kidney or spleen, beam attenuation, and poor visualization of intrahepatic structures. Each feature was scored from 0 (negative) to 2 (definite positive), yielding a total score of 0–6. Scores of 1–2, 3–4, and 5–6 indicated mild, moderate, and severe steatosis, respectively, whereas scores of 0 indicated the absence of hepatic steatosis (34, 35).

### 2.5 Statistical analysis

Continuous variables were expressed as means ± standard deviation and compared using Student’s t-test. Categorical variables were presented as frequencies (percentages) and analyzed using the chi-square test. All variables were stratified according to sex. Logistic regression analysis was performed to assess the association between MASLD status and ALT-derived markers. Multivariable logistic regression analyses were conducted with adjustments for age, sex, BMI, and TG in the total population, while age, BMI, and TG were adjusted for in sex-stratified analyses. To verify the robustness of these comparisons between the two distinct cohorts and minimize potential bias, 5-fold cross-validation was additionally performed within each dataset to assess the reproducibility of the AUC, sensitivity, and specificity for each parameter. Optimal cutoff values were derived from the NHANES dataset using Youden’s index, and receiver operating characteristic (ROC) curve analyses were conducted to evaluate the diagnostic performance of each parameter in predicting MASLD across the two cohorts.

To further enhance clinical applicability, additional cutoff values were derived, with ALT evaluated in increments of 1.0 U/L and ALT/AST and ALT/HDL evaluated in increments of 0.1. For each marker, a lower cutoff yielding the highest sensitivity while maintaining specificity above 0.5 was determined to rule in MASLD, and a higher cutoff yielding the highest specificity while maintaining sensitivity above 0.5 was determined to rule out MASLD. Pairwise comparisons of areas under the ROC curves (AUCs) were performed using DeLong’s method. To assess the relative diagnostic adequacy of ALT and its derived markers, their predictive performances were further compared with those of established composite indices currently used for hepatic steatosis screening, such as TG/HDL and HSI, in both the NHANES and real-world clinical cohorts. Considering that abdominal ultrasonography may have relatively limited sensitivity for detecting hepatic steatosis, we additionally evaluated the diagnostic performance of ALT and its derived markers against the most widely accepted pediatric ALT cutoff values (>26 U/L in males and >22 U/L in females), and the corresponding sensitivity, specificity, and other predictive indices were compared accordingly (36). Cutoff-specific MASLD prevalence was independently illustrated for each cohort and further stratified by sex, with visualizations presented as bar graphs. Power analysis was additionally performed, assuming a reference AUC of 0.6, to confirm the statistical adequacy of the sex-stratified analyses in this dataset. Statistical significance was set at p < 0.05. 3. All statistical analyses were performed using SAS version 9.4 (SAS Institute Inc., Cary, NC, USA) and R version 4.4.1 (R Foundation for Statistical Computing, Vienna, Austria).

## 3 Results

### 3.1 Baseline characteristics

**Table 1** summarizes the baseline characteristics of participants in the NHANES and real-world clinical datasets. In the NHANES cohort, males had higher height SDS, systolic BP, AST, ALT, ALT/AST, ALT/HDL, and TG/HDL levels than females, whereas total cholesterol, HDL levels, and HSI were lower in males. In the real-world clinical cohort, males also exhibited significantly higher WC, systolic BP, AST, ALT, ALT/AST, ALT/HDL, TG/HDL, and HSI levels than females.

**Table 1:** Baseline characteristics of participants in NHANES and real-world clinic data.

Variables	NHANES				Real-world clinic data				*p* value**
	Total (n=1,158)	Male (n=613)	Female (n=545)	*p* value*	Total (n=263)	Male (n=136)	Female (n=127)	*p* value*	
Age, year	15.02 ± 1.95	15.00 ± 1.97	15.05 ± 1.94	0.711	14.03 ± 4.02	14.23 ± 3.89	13.81 ± 4.16	0.395	<0.001
Height SDS	0.02 ± 1.05	0.14 ± 1.07	-0.12 ± 1.01	<0.001	0.56 ± 1.16	0.48 ± 1.08	0.64 ± 1.24	0.271	<0.001
Weight SDS	0.76 ± 1.21	0.76 ± 1.27	0.76 ± 1.13	0.911	1.14 ± 1.65	1.14 ± 1.72	1.14 ± 1.57	0.980	<0.001
BMI SDS	0.76 ± 1.16	0.70 ± 1.23	0.84 ± 1.08	0.050	1.22 ± 1.88	1.26 ± 1.90	1.17 ± 1.86	0.685	<0.001
BMI category, %				0.059				0.814	<0.001
Normal	644 (55.61)	359 (58.56)	285 (52.29)		109 (41.44)	58 (42.65)	51 (40.16)		
Overweight	210 (18.13)	98 (15.99)	112 (20.55)		28 (10.65)	13 (9.56)	15 (11.81)		
Obesity	304 (26.25)	156 (25.45)	148 (27.16)		126 (47.91)	65 (47.79)	61 (48.03)		
WC, cm	83.27 ± 16.03	83.26 ± 16.69	83.27 ± 15.27	0.987	78.47 ± 12.17	81.95 ± 12.85	74.74 ± 10.21	<0.001	<0.001
Central obesity, %	215 (18.57)	117 (19.09)	98 (17.98)	0.629	118 (44.87)	60 (44.12)	58 (45.67)	0.800	<0.001
Systolic BP, mmHg	108.63 ± 9.80	111.99 ± 9.94	104.85 ± 8.14	<0.001	114.72 ± 12.67	118.10 ± 12.64	111.11 ± 11.72	<0.001	<0.001
Diastolic BP, mmHg	64.28 ± 8.12	64.09 ± 8.55	64.49 ± 7.61	0.394	65.22 ± 11.46	66.20 ± 12.13	64.17 ± 10.63	0.151	0.210
Glucose, mg/dL	89.14 ± 16.08	90.23 ± 17.92	87.92 ± 13.63	0.013	93.54 ± 22.38	92.85 ± 6.61	94.27 ± 31.52	0.621	0.003
Total cholesterol, mg/dL	153.49 ± 29.83	149.60 ± 28.66	157.87 ± 30.52	<0.001	171.37 ± 26.61	169.66 ± 24.25	173.20 ± 28.91	0.285	<0.001
HDL, mg/dL	51.48 ± 11.82	49.17 ± 11.74	54.07 ± 11.37	<0.001	52.85 ± 10.34	51.78 ± 9.93	53.99 ± 10.69	0.083	0.060
TG, mg/dL	92.41 ± 53.95	94.80 ± 60.16	89.72 ± 45.89	0.104	96.68 ± 44.86	99.32 ± 52.00	93.85 ± 35.67	0.318	0.181
AST, U/L	19.69 ± 8.45	21.73 ± 10.50	17.40 ± 4.23	<0.001	26.22 ± 17.34	28.63 ± 20.93	23.64 ± 11.93	0.018	<0.001
ALT, U/L	16.16 ± 11.07	18.83 ± 13.51	13.17 ± 6.19	<0.001	29.43 ± 35.14	35.39 ± 42.17	23.06 ± 24.14	0.004	<0.001
ALT/AST	0.8 ± 0.32	0.85 ± 0.37	0.75 ± 0.25	<0.001	0.98 ± 0.46	1.08 ± 0.51	0.88 ± 0.39	<0.001	<0.001
ALT/HDL	0.34 ± 0.30	0.42 ± 0.38	0.26 ± 0.15	<0.001	0.60 ± 0.75	0.73 ± 0.89	0.47 ± 0.53	0.004	<0.001
TG/HDL	2.00 ± 1.66	2.18 ± 1.98	1.79 ± 1.17	<0.001	1.99 ± 1.22	2.09 ± 1.40	1.88 ± 1.00	0.157	0.924
HSI	32.16 ± 8.25	31.08 ± 8.44	33.38 ± 7.86	<0.001	32.81 ± 7.37	33.26 ± 7.94	32.32 ± 6.69	0.302	0.214
CAP, dB/m	220.29 ± 53.33	223.09 ± 54.30	217.14 ± 52.08	0.058	–	–	–	–	
MASLD (%)	284 (24.53)	161 (26.26)	123 (22.57)	0.145	95 (36.12)	55 (40.44)	40 (31.50)	0.131	<0.001

Continuous variables are presented as mean ± standard deviation, and categorical data as numbers (percentages).

*P values were assessed using Student’s t-test for continuous variables and the chi-square test for categorical variables.

**P values were calculated by comparing NHANES data and real-world clinic data.

NHANES, National Health and Nutrition Examination Survey; SDS, standard deviation score; BMI, body mass index; WC, waist circumference; BP, blood pressure; AST, aspartate aminotransferase; ALT, alanine aminotransferase; HDL, high-density lipoprotein cholesterol; TG, triglyceride; HSI, hepatic steatosis index; CAP, controlled attenuation parameter; MASLD, metabolic dysfunction-associated steatotic liver disease.

### 3.2 Logistic regression analysis for predicting MASLD using ALT and ALT-derived markers

Univariable logistic regression analyses demonstrated that ALT, ALT/AST, ALT/HDL, TG/HDL, and HSI were significantly and positively associated with MASLD in the total, male, and female groups across both the NHANES and real-world clinical datasets (all p < 0.05) (**Table 2**). These associations remained robust after adjusting for age, sex, BMI, and TG in the total population, and for age, BMI, and TG in the sex-stratified analyses, with significant positive associations between all markers and MASLD observed across nearly all subgroups in both cohorts (all p < 0.05), with the exception of TG/HDL in the male subgroup of the real-world clinical dataset (p = 0.316).

**Table 2:** Logistic regression analysis for MASLD using parameters from NHANES and real-world clinic data.

Variable	OR (95% CI)	*p* value	Adjusted OR (95% CI)*	*p* value
NHANES				
Total				
ALT	1.09 (1.07-1.10)	<0.001	1.04 (1.02-1.06)	<0.001
ALT/AST	1.38 (1.31-1.46)	<0.001	1.15 (1.08-1.22)	<0.001
ALT/HDL	1.49 (1.39-1.60)	<0.001	1.19 (1.10-1.29)	<0.001
TG/HDL	1.68 (1.51 – 1.87)	<0.001	1.29 (1.14-1.46)	<0.001
HSI	1.27 (1.23-1.31)	<0.001	1.19 (1.10-1.28)	<0.001
Male				
ALT	1.08 (1.06-1.10)	<0.001	1.03(1.00-1.05)	0.017
ALT/AST	1.36 (1.28-1.46)	<0.001	1.13(1.04-1.22)	0.003
ALT/HDL	1.45 (1.34-1.58)	<0.001	1.14(1.04-1.26)	0.006
TG/HDL	1.58 (1.39-1.79)	<0.001	1.17 (1.00-1.37)	0.047
HSI	1.35 (1.28-1.42)	<0.001	1.17 (1.06-1.29)	0.002
Female				
ALT	1.13 (1.09-1.17)	<0.001	1.07 (1.03-1.12)	0.001
ALT/AST	1.43 (1.30-1.56)	<0.001	1.18 (1.06-1.31)	0.002
ALT/HDL	1.94 (1.64-2.30)	<0.001	1.37 (1.12-1.66)	0.002
TG/HDL	1.89 (1.57-2.28)	<0.001	1.51 (1.22-1.89)	<0.001
HSI	1.24 (1.19-1.29)	<0.001	1.21 (1.06-1.38)	0.004
Real-world clinic data				
Total				
ALT	1.08 (1.06-1.11)	<0.001	1.07 (1.04-1.10)	<0.001
ALT/AST	1.44 (1.31-1.59)	<0.001	1.31 (1.17-1.48)	<0.001
ALT/HDL	1.44 (1.29-1.62)	<0.001	1.32 (1.16-1.50)	<0.001
TG/HDL	2.45 (1.84-3.28)	<0.001	1.69(1.23-2.33)	0.001
HSI	1.49 (1.35-1.64)	<0.001	1.42 (1.22-1.66)	<0.001
Male				
ALT	1.07 (1.04-1.10)	<0.001	1.07 (1.03-1.12)	<0.001
ALT/AST	1.37 (1.22-1.55)	<0.001	1.30 (1.12-1.52)	<0.001
ALT/HDL	1.36 (1.18-1.56)	<0.001	1.39 (1.15-1.68)	<0.001
TG/HDL	1.70 (1.26-2.31)	<0.001	1.19 (0.84-1.69)	0.316
HSI	1.51 (1.31-1.74)	<0.001	1.40 (1.16-1.70)	<0.001
Female				
ALT	1.11 (1.06-1.16)	<0.001	1.08 (1.03-1.14)	0.003
ALT/AST	1.58 (1.33-1.88)	<0.001	1.33 (1.09-1.62)	0.005
ALT/HDL	1.61 (1.30-1.99)	<0.001	1.30 (1.07-1.58)	0.008
TG/HDL	5.46 (2.98-10.03)	<0.001	3.90 (1.93-7.85)	<0.001
HSI	1.50 (1.29-1.73)	<0.001	1.46 (1.13-1.89)	0.004

Odds ratios and 95% confidence intervals for each parameter are presented with corresponding p-values for both the unadjusted and adjusted models.

*Adjusted for age, sex, BMI, and TG in the total group, and for age, BMI, and TG in the male and female group. For the TG/HDL ratio variable, adjustment for TG was omitted in the adjusted model.

MASLD, metabolic dysfunction-associated steatotic liver disease; ALT, alanine aminotransferase; NHANES, National Health and Nutrition Examination Survey; OR, odds ratio; CI, confidence interval; AST, aspartate aminotransferase; TG, triglyceride; HDL, high-density lipoprotein cholesterol; HSI, hepatic steatosis index.

### 3.3 Optimal cutoff values and ROC curve analysis for predicting MASLD

**Figure 2:**
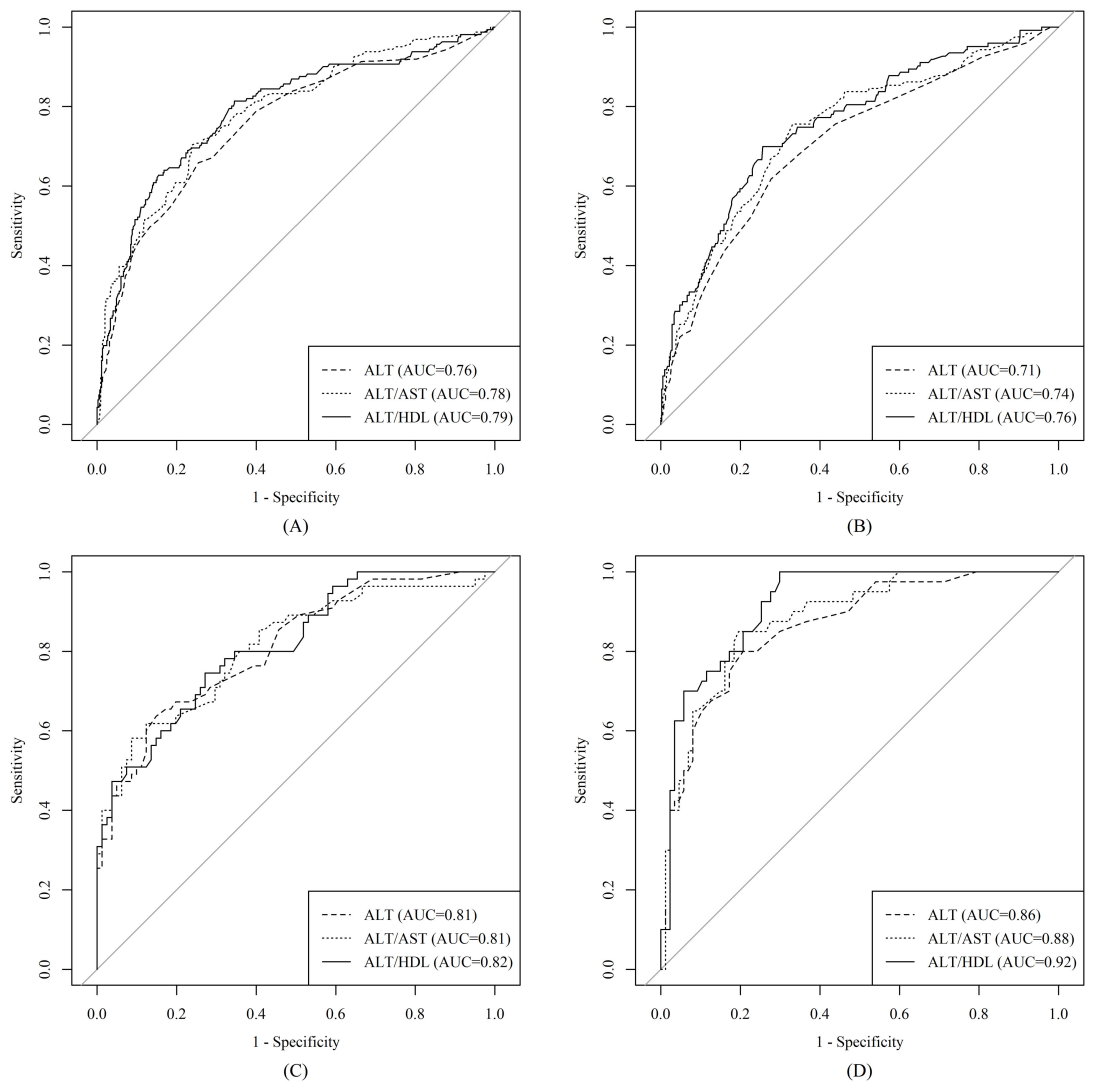
ROC curves of ALT and its derived markers for predicting MASLD. **(A)** NHANES male participants. **(B)** NHANES female participants. **(C)** Real-world clinic data, male participants. **(D)** Real-world clinic data, female participants. ROC, receiver operating characteristic; ALT, alanine aminotransferase; MASLD, metabolic dysfunction-associated steatotic liver disease; NHANES, National Health and Nutrition Examination Survey; AST, aspartate aminotransferase; HDL, high-density lipoprotein cholesterol.

In ROC curve analysis using NHANES data, the AUCs of ALT, ALT/AST, ALT/HDL, TG/HDL, and HSI were 0.73, 0.77, 0.76, 0.72, and 0.90, respectively, for all participants (**Table 3**, **Figure 2**). In sex-specific analyses, the corresponding values were 0.76, 0.78, 0.79, 0.72, and 0.92 in males, and 0.71, 0.74, 0.76, 0.70, and 0.89 in females, respectively. In pairwise comparisons, the AUCs of ALT/HDL and HSI were significantly higher than those for ALT and TG/HDL in the total, male, and female groups in the NHANES cohort (all p < 0.001) (**Supplementary Table 1**). In addition, the AUC for ALT/AST was significantly higher than that for ALT alone in the total group.

**Table 3:** Diagnostic performance and cutoff values of the parameters for MASLD in NHANES and real-world clinic data.

	AUC (95% CI)	*p* value	Cutoff*	Sensitivity (95% CI)	Specificity (95% CI)	Accuracy	PPV	NPV
NHANES								
Total								
ALT	0.73 (0.69-0.77)	<0.001	>14.500	0.67 (0.62-0.73)	0.68 (0.65-0.72)	0.68 (0.65-0.71)	0.41 (0.36-0.45)	0.87 (0.84-0.89)
ALT	0.73 (0.69-0.77)	<0.001	>26.000 in male; >22.000 in female	0.32 (0.26-0.37)	0.93 (0.92-0.95)	0.78 (0.76-0.81)	0.61 (0.53-0.69)	0.81 (0.78-0.83)
ALT/AST	0.77 (0.73-0.80)	<0.001	>0.738	0.79 (0.74-0.84)	0.63 (0.60-0.66)	0.67 (0.64-0.70)	0.41 (0.37-0.45)	0.90 (0.88-0.93)
ALT/HDL	0.76 (0.73-0.80)	<0.001	>0.291	0.72 (0.67-0.77)	0.69 (0.66-0.72)	0.70 (0.67-0.73)	0.43 (0.39-0.48)	0.88 (0.86-0.91)
TG/HDL	0.72 (0.68-0.75)	<0.001	>1.511	0.75 (0.70-0.80)	0.56 (0.53-0.59)	0.61 (0.58-0.63)	0.36 (0.32-0.39)	0.87 (0.85-0.90)
HSI	0.90 (0.88-0.92)	<0.001	>33.740	0.84 (0.80-0.88)	0.84 (0.82-0.87)	0.84 (0.82-0.86)	0.63 (0.59-0.68)	0.94 (0.93-0.96)
Male								
ALT	0.76 (0.71-0.80)	<0.001	>17.500	0.66 (0.59-0.73)	0.75 (0.71-0.79)	0.72 (0.69-0.76)	0.48 (0.41-0.55)	0.86 (0.83-0.89)
ALT	0.76 (0.71-0.80)	<0.001	>26.000	0.39 (0.32-0.47)	0.92 (0.89-0.94)	0.78 (0.75-0.81)	0.63 (0.54-0.72)	0.81 (0.77-0.84)
ALT/AST	0.78 (0.74-0.83)	<0.001	>0.860	0.70 (0.63-0.77)	0.76 (0.72-0.80)	0.75 (0.71-0.78)	0.51 (0.45-0.58)	0.88 (0.85-0.91)
ALT/HDL	0.79 (0.75-0.83)	<0.001	>0.426	0.63 (0.55-0.70)	0.85 (0.81-0.88)	0.79 (0.76-0.82)	0.59 (0.52-0.66)	0.86 (0.83-0.90)
TG/HDL	0.72 (0.68-0.77)	<0.001	>1.760	0.68 (0.60-0.75)	0.66 (0.62-0.70)	0.66 (0.63-0.70)	0.41 (0.35-0.47)	0.85 (0.81-0.89)
HSI	0.92 (0.90-0.95)	<0.001	>31.050	0.93 (0.89-0.97)	0.80 (0.76-0.83)	0.83 (0.80-0.86)	0.62 (0.56-0.68)	0.97 (0.95-0.99)
Female								
ALT	0.71 (0.65-0.76)	<0.001	>13.500	0.62 (0.53-0.70)	0.72 (0.68-0.77)	0.70 (0.66-0.74)	0.39 (0.32-0.46)	0.87 (0.83-0.90)
ALT	0.71 (0.65-0.76)	<0.001	>22.000	0.22 (0.15-0.29)	0.95 (0.93-0.97)	0.79 (0.75-0.82)	0.57 (0.43-0.72)	0.81 (0.77-0.84)
ALT/AST	0.74 (0.69-0.79)	<0.001	>0.745	0.76 (0.68-0.83)	0.67 (0.62-0.71)	0.69 (0.65-0.73)	0.40 (0.34-0.46)	0.90 (0.87-0.94)
ALT/HDL	0.76 (0.71-0.81)	<0.001	>0.260	0.70 (0.62-0.78)	0.74 (0.70-0.79)	0.73 (0.70-0.77)	0.44 (0.37-0.51)	0.89 (0.86-0.93)
TG/HDL	0.70 (0.65-0.76)	<0.001	>1.545	0.70 (0.62-0.78)	0.60 (0.55-0.65)	0.62 (0.58-0.66)	0.34 (0.28-0.40)	0.87 (0.83-0.91)
HSI	0.89 (0.86-0.92)	<0.001	>33.861	0.89 (0.83-0.94)	0.78 (0.74-0.82)	0.80 (0.77-0.84)	0.54 (0.47-0.61)	0.96 (0.94-0.98)
Real-world clinic data								
Total								
ALT	0.84 (0.79-0.89)	<0.001	>14.500	0.89 (0.83-0.96)	0.52 (0.45-0.60)	0.66 (0.60-0.72)	0.52 (0.44-0.59)	0.90 (0.84-0.96)
ALT	0.84 (0.79-0.89)	<0.001	>26.000 in male; >22.000 in female	0.65 (0.56-0.75)	0.86 (0.80-0.91)	0.78 (0.73-0.83)	0.72 (0.63-0.82)	0.81 (0.76-0.87)
ALT/AST	0.84 (0.80-0.89)	<0.001	>0.738	0.92 (0.86-0.97)	0.52 (0.44-0.59)	0.66 (0.60-0.72)	0.52 (0.44-0.59)	0.92 (0.86-0.97)
ALT/HDL	0.86 (0.82-0.91)	<0.001	>0.291	0.93 (0.87-0.98)	0.58 (0.51-0.66)	0.71 (0.65-0.76)	0.56 (0.48-0.63)	0.93 (0.89-0.98)
TG/HDL	0.77 (0.70-0.83)	<0.001	>1.511	0.78 (0.70-0.86)	0.57 (0.50-0.65)	0.65 (0.59-0.70)	0.51 (0.43-0.59)	0.82 (0.75-0.89)
HSI	0.92 (0.89-0.96)	<0.001	>33.740	0.83 (0.76-0.91)	0.85 (0.79-0.90)	0.84 (0.80-0.88)	0.75 (0.67-0.83)	0.90 (0.85-0.95)
Male								
ALT	0.81 (0.74-0.88)	<0.001	>17.500	0.85 (0.76-0.95)	0.54 (0.43-0.65)	0.67 (0.59-0.75)	0.56 (0.45-0.67)	0.85 (0.75-0.94)
ALT	0.81 (0.74-0.88)	<0.001	>26.000	0.65 (0.53-0.78)	0.81 (0.73-0.90)	0.75 (0.68-0.82)	0.71 (0.58-0.83)	0.78 (0.69-0.87)
ALT/AST	0.81 (0.74-0.89)	<0.001	>0.860	0.84 (0.74-0.93)	0.59 (0.49-0.70)	0.69 (0.61-0.77)	0.58 (0.47-0.69)	0.84 (0.75-0.94)
ALT/HDL	0.82 (0.74-0.89)	<0.001	>0.426	0.75 (0.63-0.86)	0.69 (0.59-0.79)	0.71 (0.64-0.79)	0.62 (0.50-0.74)	0.80 (0.71-0.89)
TG/HDL	0.71(0.62-0.79)	0.031	>1.760	0.69 (0.57-0.81)	0.64 (0.54-0.75)	0.66 (0.58-0.74)	0.57 (0.45-0.69)	0.75 (0.65-0.86)
HSI	0.93(0.89-0.97)	<0.001	>31.050	0.96 (0.91-1.00)	0.73 (0.63-0.83)	0.82 (0.76-0.89)	0.71 (0.60-0.81)	0.97 (0.92-1.00)
Female								
ALT	0.86 (0.79-0.93)	<0.001	>13.500	0.90 (0.81-0.99)	0.53 (0.42-0.63)	0.65 (0.56-0.73)	0.47 (0.36-0.58)	0.92 (0.84-1.00)
ALT	0.86 (0.79-0.93)	<0.001	>22.000	0.65 (0.50-0.80)	0.90 (0.83-0.96)	0.82 (0.75-0.89)	0.74 (0.60-0.89)	0.85 (0.77-0.92)
ALT/AST	0.88 (0.82-0.94)	<0.001	>0.745	0.90 (0.81-0.99)	0.64 (0.54-0.74)	0.72 (0.65-0.80)	0.54 (0.42-0.66)	0.93 (0.87-1.00)
ALT/HDL	0.92 (0.87-0.96)	<0.001	>0.260	1.00 (1.00-1.00)	0.61 (0.51-0.71)	0.73 (0.66-0.81)	0.54 (0.43-0.65)	1.00 (1.00-1.00)
TG/HDL	0.84 (0.76-0.92)	<0.001	>1.545	0.83 (0.71-0.94)	0.63 (0.53-0.73)	0.69 (0.61-0.77)	0.51 (0.39-0.63)	0.89 (0.81-0.97)
HSI	0.93 (0.88-0.97)	<0.001	>33.861	0.90 (0.81-0.99)	0.83 (0.75-0.91)	0.85 (0.79-0.91)	0.71 (0.58-0.83)	0.95 (0.90-1.00)

Cutoff values optimize sensitivity and specificity. AUC indicates the area under the ROC curve for each parameter.

*Cutoff values were derived from NHANES data and applied to real-world clinical data.

ALT, alanine aminotransferase; MASLD, metabolic dysfunction-associated steatotic liver disease; NHANES, National Health and Nutrition Examination Survey; AUC, area under the curve; CI, confidence interval; PPV, positive predictive value; NPV, negative predictive value; AST, aspartate aminotransferase; TG, triglyceride; HDL, high-density lipoprotein cholesterol; HSI, hepatic steatosis index.

The optimal cutoff values for predicting MASLD were determined as >14.50 for ALT, >0.74 for ALT/AST, and >0.29 for ALT/HDL in the total population (**Table 3**). In males, the corresponding values were >17.50, >0.86, and >0.43, whereas in females they were >13.50, >0.75, and >0.26, respectively. Compared with the currently established ALT cutoff values (>26 U/L in males and >22 U/L in females), the optimal cutoff values for ALT, ALT/AST, and ALT/HDL identified in this study demonstrated higher sensitivity across all subgroups. In addition, the higher cutoff values (yielding the highest specificity) were determined as >17.0 for ALT, >0.9 for ALT/AST, and >0.4 for ALT/HDL in the total population; >20.0, >1.0, and >0.5 in males; and >14.0, >0.8, and >0.3 in females, respectively (**Supplementary Table 2**). The corresponding lower cutoff values (yielding the highest sensitivity) were >12.0, >0.7, and >0.3 in the total population; >13.0, >0.7, and >0.3 in males; and >11.0, >0.7, and >0.2 in females, respectively.

In the real-world clinical cohort, the AUCs of ALT, ALT/AST, ALT/HDL, TG/HDL, and HSI were 0.84, 0.84, 0.86, 0.77, and 0.92, respectively, in the total population (**Table 3**, **Figure 2**). In sex-specific analyses, the corresponding values were 0.81, 0.81, 0.82, 0.71 and 0.93 in males and 0.86, 0.88, 0.92, 0.84, and 0.93 in females, respectively. In pairwise comparisons, the AUC for ALT/HDL was significantly higher than that for ALT in all subgroups (**Supplementary Table 1**).

The 5-fold cross-validation demonstrated consistent statistical significance across all analyses, with AUC values comparable to those obtained in the original analysis (**Supplementary Table 3**). The power analysis confirmed that statistical power exceeded 0.9 for ALT, ALT/AST, and ALT/HDL across all subgroups, supporting the statistical adequacy of the sex-stratified analyses in the real-world clinical cohort.

### 3.4 Proportion of MASLD according to the cutoff values of each marker

**Figure 3:**
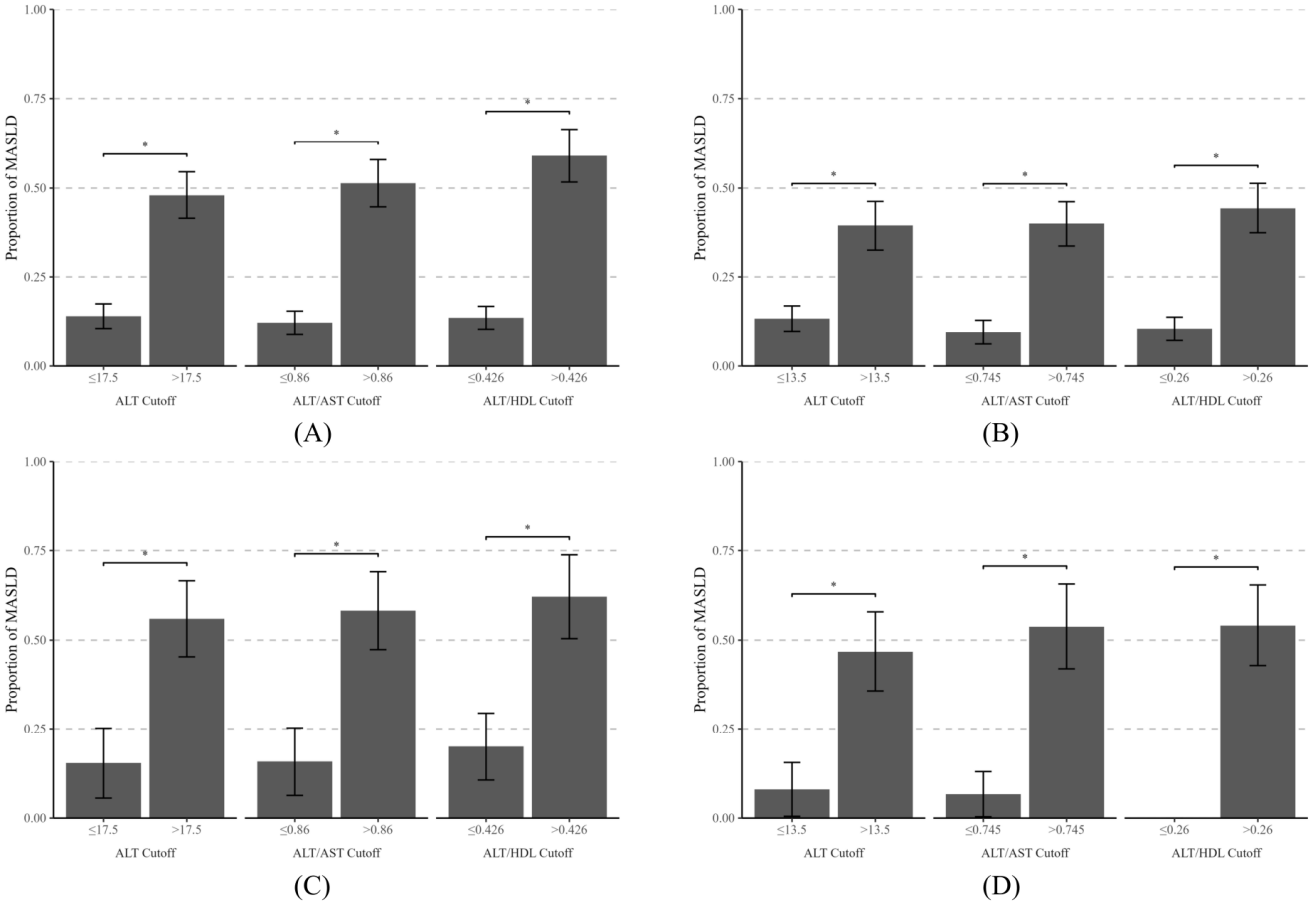
Proportion of participants with MASLD relative to the cutoff points of each parameter. **(A)** NHANES male participants. **(B)** NHANES female participants. **(C)** Real-world clinic data, male participants. **(D)** Real-world clinic data, female participants. MASLD, metabolic dysfunction-associated steatotic liver disease; NHANES, National Health and Nutrition Examination Survey; ALT, alanine aminotransferase; AST, aspartate aminotransferase; HDL, high-density lipoprotein cholesterol.

**Figure 3** shows the distribution of MASLD prevalence according to the cutoff values for each marker across both datasets. In both males and females, participants with marker values above the established cutoffs exhibited significantly higher proportions of MASLD in both the NHANES dataset (**Figures 3A, B**) and the real-world clinical cohort (**Figures 3C, D**).

## 4 Discussion

In the present study, ALT and its derived markers demonstrated significant associations with MASLD in both the NHANES cohort and the real-world clinical cohort, even after adjusting for age, sex, BMI, and TG. Optimal cutoff values, as well as higher and lower cutoff values, were determined using the NHANES data according to sex and subsequently applied to both cohorts to evaluate diagnostic performance via ROC curve analysis. All three markers showed acceptable predictive ability, with ALT/HDL exhibiting the highest AUC, followed by ALT/AST and ALT. This trend was consistent across the total population and sex-specific subgroups. Moreover, the proportion of participants with MASLD differed markedly depending on whether their marker values were above or below the established cutoffs, further supporting the discriminative utility of these thresholds.

While pediatric NAFLD guidelines recommend ALT as a primary screening tool for pediatric NAFLD, our study further confirmed its utility in predicting the newly defined entity, MASLD, which emphasizes the metabolic aspects of hepatic steatosis (12). The mechanism underlying ALT elevation in NAFLD, a widely recognized form of hepatic steatosis, involves the accumulation of fatty acids within hepatocytes, leading to lipotoxicity. This process induces endoplasmic reticulum stress, impairs the unfolded protein response, activates inflammasomes, and initiates apoptotic pathways. Consequently, hepatocellular injury occurs, leading to the release of ALT into the bloodstream (37, 38).

For the optimal cutoff value of ALT for chronic liver disease in children, a US study recommended 25.8 U/L in males and 22.1 U/L in females, defined by the 95th percentile, as the upper normal limit (UNL) (14, 15). In a Korean study, the UNL of ALT, defined by the 95th percentile, was identified as 24.1 U/L in boys and 17.7 U/L in girls (20). Although these cutoffs have been widely used, they were fundamentally derived based on population-defined ULN rather than established as thresholds specifically intended to predict MASLD. Thus, we identified optimal cutoffs for detecting pediatric MASLD, which were 17.5 IUL in males and 13.5 IU/L in females; these values were lower than previously applied ALT ULNs. From a public health perspective, this finding has meaningful implications because the primary objective of population-level screening is to minimize missed cases rather than to maximize specificity. The cutoff values identified in this study, each demonstrating higher sensitivity than the currently established ALT thresholds, may represent more suitable screening criteria for detecting MASLD in children and adolescents.

Although AST is a key component of liver function tests alongside ALT, its clinical specificity is lower than that of ALT due to its presence in various extrahepatic tissues, including muscle, kidney, and brain (13). Nanji et al. were the first to propose the ALT/AST ratio as a marker for assessing the degree of hepatic steatosis in patients with morbid obesity (22). This proposal is primarily based on the differences in the intracellular distribution and serum half-life of AST and ALT. AST is found in both the mitochondria and the cytosol, whereas ALT is predominantly located in the cytosol. In addition, AST has a shorter serum half-life than ALT. Consequently, in chronic liver injuries such as hepatic steatosis, ALT levels tend to remain elevated, leading to an increased ALT/AST ratio (25). A US study reported that the optimal cutoff value of the ALT/AST ratio for NAFLD was 0.918 among youth (24). In the present study, the corresponding ALT/AST ratio cutoffs for MASLD were 0.738 for the total population, 0.860 for males, and 0.745 for females, respectively.

In our study, the ALT/HDL ratio demonstrated better predictive performance for MASLD than ALT alone. Originally proposed as a predictor for diabetes, the ALT/HDL ratio has also shown potential as a useful marker for identifying metabolic liver diseases, including NAFLD (23). The ALT/HDL ratio combines ALT, a sensitive marker of hepatic fat accumulation, with HDL, an indicator of reverse cholesterol transport, which is often impaired in hepatic steatosis. HDL plays a pivotal metabolic role by facilitating reverse cholesterol transport, and its metabolism is intricately regulated by factors such as insulin sensitivity, lipid remodeling, and cellular cholesterol efflux (39). Given its clinical relevance, HDL is widely recognized as a key marker of metabolic disease. Both its absolute concentration and derived ratios, such as TG/HDL and uric acid/HDL, have been extensively investigated in clinical studies (40, 41). In a Japanese study, the optimal cutoff value of ALT/HDL for NAFLD was 0.413 among adults (23). In the present study, the optimal ALT/HDL cutoff values for pediatric MASLD were 0.291 for the total population, 0.426 for males, and 0.260 for females. These findings indicate that distinct ALT/HDL cutoffs may be required not only between adults and children, but also between NAFLD and MASLD, to enhance diagnostic accuracy.

Compared to previously established composite markers, ALT and its derived markers generally outperformed the TG/HDL ratio across both cohorts. Although the HSI, a well-recognized indicator of hepatic steatosis, demonstrated high AUC values, its broader clinical applicability remains limited by the requirement for additional anthropometric input and a more complex calculation formula (42). In contrast, ALT and its derived markers rely solely on routinely measured liver enzymes and a single lipid parameter, offering a more intuitive and readily implementable approach for use in both individual clinical practice and large-scale public health screening settings (36). These considerations emphasize the practical utility of ALT-derived markers as accessible screening tools, even in contexts where more computationally complex indices, such as HSI, may demonstrate numerically superior discriminative performance.

Our study had several limitations. First, it was a retrospective and cross-sectional study, which limited causal inference. Second, the real-world clinical dataset had a smaller sample size than the NHANES dataset, potentially reducing the statistical power of the subgroup analyses and limiting the generalizability of the findings although a power analysis confirmed adequate statistical power for the sex-stratified comparisons in this dataset; therefore, the sex-stratified findings in the real-world clinical cohort should be regarded as exploratory in nature, and further validation in larger pediatric cohorts is warranted. Third, different diagnostic methods were used to define MASLD in the two cohorts.

Despite these limitations, the 5-fold cross-validation yielded closely comparable AUC values and statistical significance across all analyses, further supporting the robustness of the findings within each cohort and reinforcing their generalizability. Furthermore, to address the discrepancy arising from the use of different diagnostic methods, we compared the optimal cutoffs with classical cutoffs and suggested lower and higher cutoffs for markers to overcome the limited sensitivity of ultrasonography. As a result, the diagnostic utility of ALT-derived markers for MASLD was consistently demonstrated using both CAP and ultrasonography, thereby strengthening the robustness of our findings. In addition, the validity of these markers was confirmed not only in a large-scale population-based dataset, but also in a real-world clinical cohort with distinct characteristics, thereby supporting the broader applicability of our results across diverse clinical settings.

In conclusion, this study evaluated the diagnostic performance and optimal cutoffs of ALT and its derived markers for predicting pediatric MASLD using both population-based and real-world clinical data. All three markers showed significant associations with MASLD, with the ALT/HDL ratio demonstrating the highest predictive accuracy across both cohorts and in sex-stratified analyses. ALT and its derived markers are based on simple, routinely available laboratory tests, making them practical and cost-effective tools for early, non-invasive screening in clinical settings. The superior performance of ALT/HDL over ALT alone underscores the added value of the composite indices that reflect both hepatic injury and metabolic status. These findings support the broad clinical utility of ALT-derived markers for pediatric MASLD screening, although prospective studies are needed to validate their use in diverse populations.

## Data Availability

The datasets presented in this study can be found in online repositories. The names of the repository/repositories and accession number(s) can be found below: https://wwwn.cdc.gov/nchs/nhanes/.

## References

[1] Rinella ME Lazarus JV Ratziu V Francque SM Sanyal AJ Kanwal F . A multisociety Delphi consensus statement on new fatty liver disease nomenclature. Hepatology. (2023) 78:1966–86. doi: 10.1097/hep.0000000000000520 37363821 PMC10653297

[2] Schwärzler J Felix G Christoph G E. AT Tilg H . The pathophysiology of MASLD: an immunometabolic perspective. Expert Rev Clin Immunol. (2024) 20:375–86. doi: 10.1080/1744666X.2023.2294046 38149354

[3] Targher G Byrne CD Tilg H . MASLD: a systemic metabolic disorder with cardiovascular and Malignant complications. Gut. (2024) 73:691–702. doi: 10.1136/gutjnl-2023-330595 38228377

[4] Bansal SK Bansal MB . Pathogenesis of MASLD and MASH–role of insulin resistance and lipotoxicity. Alimentary Pharmacol Ther. (2024) 59:S10–22. doi: 10.1111/apt.17930 38451123

[5] Ntikoudi A Papachristou A Tsalkitzi A Margari N Evangelou E Vlachou E . Metabolic-associated steatotic liver disease (MASLD) and type 2 diabetes: mechanisms, diagnostic approaches, and therapeutic interventions. Diabetology. (2025) 6:23. doi: 10.3390/diabetology6040023 30654563

[6] Kalligeros M Vassilopoulos A Vassilopoulos S Victor DW Mylonakis E Noureddin M . Prevalence of steatotic liver disease (MASLD, metALD, and ALD) in the United States: NHANES 2017-2020. Clin Gastroenterol Hepatol. (2024) 22:1330–2:e4. doi: 10.1016/j.cgh.2023.11.003 37949334

[7] Miao L Targher G Byrne CD Cao Y-Y Zheng M-H . Current status and future trends of the global burden of MASLD. Trends Endocrinol Metab. (2024) 35:697–707. doi: 10.1016/j.tem.2024.02.007 38429161

[8] Sun M Sun H . Recent prevalence and trends of obesity and metabolic dysfunction‐associated steatotic liver disease (MASLD) among US adolescents: 1999 to 2020. Pediatr Obes. (2025) 20:e70003. doi: 10.1111/ijpo.70003 39967492

[9] Le P Tatar M Dasarathy S Alkhouri N Herman WH Taksler GB . Estimated burden of metabolic dysfunction–associated steatotic liver disease in US adults, 2020 to 2050. JAMA Netw Open. (2025) 8:e2454707-e. doi: 10.1001/jamanetworkopen.2024.54707 39821400 PMC11742522

[10] Simon TG Roelstraete B Hartjes K Shah U Khalili H Arnell H . Non-alcoholic fatty liver disease in children and young adults is associated with increased long-term mortality. J Hepatol. (2021) 75:1034–41. doi: 10.1016/j.jhep.2021.06.034 34224779 PMC8530955

[11] Özer Y Yapar Gümüş C . Predictors of pediatric non-alcoholic fatty liver disease (NAFLD) in obese children and adolescents: is serum ALT level sufficient in detecting NAFLD? Zeynep Kamil Med J. (2024) 55:59–66. doi: 10.14744/zkmj.2024.67209

[12] Vos MB Abrams SH Barlow SE Caprio S Daniels SR Kohli R . NASPGHAN clinical practice guideline for the diagnosis and treatment of nonalcoholic fatty liver disease in children: recommendations from the Expert Committee on NAFLD (ECON) and the North American Society of Pediatric Gastroenterology, Hepatology and Nutrition (NASPGHAN). J Pediatr Gastroenterol Nutr. (2017) 64:319–34. doi: 10.1097/mpg.0000000000001482 28107283 PMC5413933

[13] Kalas MA Chavez L Leon M Taweesedt PT Surani S . Abnormal liver enzymes: a review for clinicians. World J Hepatol. (2021) 13:1688–98. doi: 10.4254/wjh.v13.i11.1688 34904038 PMC8637680

[14] Schwimmer JB Dunn W Norman GJ Pardee PE Middleton MS Kerkar N . SAFETY Study: Alanine aminotransferase cutoff values are set too high for reliable detection of pediatric chronic liver disease. Gastroenterology. (2010) 138:1357–64:e2. doi: 10.1053/j.gastro.2009.12.052 20064512 PMC2846968

[15] Draijer LG Feddouli S Bohte AE vd Baan Slootweg O Pels Rijcken TH Benninga MA . Comparison of diagnostic accuracy of screening tests ALT and ultrasound for pediatric non-alcoholic fatty liver disease. Eur J Pediatr. (2019) 178:863–70. doi: 10.1007/s00431-019-03362-3 30903305 PMC6511345

[16] Molleston JP Schwimmer JB Yates KP Murray KF Cummings OW Lavine JE . Histological abnormalities in children with nonalcoholic fatty liver disease and normal or mildly elevated alanine aminotransferase levels. J Pediatr. (2014) 164:707–13:e3. doi: 10.1016/j.jpeds.2013.10.071 24360992 PMC3962701

[17] Song K Kwon YJ Lee E Lee HS Youn YH Baik SJ . Optimal cutoffs of fatty liver index and hepatic steatosis index in diagnosing pediatric metabolic dysfunction-associated steatotic liver disease. Clin Gastroenterol Hepatol. (2026) 24:421–31:e6. doi: 10.1016/j.cgh.2025.06.011 40582390

[18] Ting YW Jalaludin MY Zaini AA Mohamed R . Triglyceride to high-density lipoprotein cholesterol ratio is an independent predictor of liver fibrosis among pediatrics non-alcoholic fatty liver disease. Front Endocrinol (Lausanne). (2022) 13:1071350. doi: 10.3389/fendo.2022.1071350 36589844 PMC9800858

[19] Condon S Hu H Kong M Cave MC McClain CJ . ALT poorly predicts nonalcoholic fatty liver disease (NAFLD) and liver fibrosis as determined by vibration-controlled transient elastography in adult National Health and Nutrition Examination Survey 2017-2018. Am J Med Sci. (2024) 367:310–22. doi: 10.1016/j.amjms.2024.01.022 38307172 PMC11299156

[20] Kang Y Park S Kim S Koh H . Normal serum alanine aminotransferase and non-alcoholic fatty liver disease among Korean adolescents: a cross-sectional study using data from KNHANES 2010–2015. BMC Pediatr. (2018) 18:215. doi: 10.1186/s12887-018-1202-z 29976192 PMC6034238

[21] Liu Z Que S Xu J Peng T . Alanine aminotransferase-old biomarker and new concept: a review. Int J Med Sci. (2014) 11:925–35. doi: 10.7150/ijms.8951 25013373 PMC4081315

[22] Nanji AA French SW Freeman JB . Serum alanine aminotransferase to aspartate aminotransferase ratio and degree of fatty liver in morbidly obese patients. Enzyme. (2017) 36:266–9. doi: 10.1159/000469304 3569188

[23] Qiu J Kuang M Yang R Yu C He S Sheng G . The newly proposed alanine aminotransferase to high-density lipoprotein cholesterol ratio has shown effectiveness in identifying non-alcoholic fatty liver disease. Front Endocrinol. (2023) 14:1239398. doi: 10.3389/fendo.2023.1239398 37727457 PMC10505795

[24] Xuan Y Wu D Zhang Q Yu Z Yu J Zhou D . Elevated ALT/AST ratio as a marker for NAFLD risk and severity: insights from a cross-sectional analysis in the United States. Front Endocrinol. (2024) 15:1457598. doi: 10.3389/fendo.2024.1457598 39253584 PMC11381241

[25] Long MT Pedley A Colantonio LD Massaro JM Hoffmann U Muntner P . Development and validation of the Framingham steatosis index to identify persons with hepatic steatosis. Clin Gastroenterol Hepatol. (2016) 14:1172–80:e2. doi: 10.1016/j.cgh.2016.03.034 27046482 PMC4955680

[26] Bithi N Panda S Dowlin MD Devaraj S . Assessment of non-invasive fibrosis scoring systems in pediatric MASLD: a retrospective comparison across healthy, MASLD, and biopsy-staged groups. Clin Chim Acta. (2026) 586:120905. doi: 10.1016/j.cca.2026.120905 41692135

[27] Centers for Disease C Prevention . National Center for Health Statistics. Clinical growth charts: Centers for Disease Control and Prevention (2000). Available online at: https://www.cdc.gov/growthcharts/clinical_charts.htm. (Accessed March 15, 2024).

[28] Kim JH Yun S Hwang S Shim JO Chae HW Lee YJ . The 2017 Korean National Growth Charts for children and adolescents: development, improvement, and prospects. Korean J Pediatr. (2018) 61:135. doi: 10.3345/kjp.2018.61.5.135 29853938 PMC5976563

[29] Kim MY An S Shim YS Lee HS Hwang JS . Waist-height ratio and body mass index as indicators of obesity and cardiometabolic risk in Korean children and adolescents. Ann Pediatr Endocrinol Metab. (2024) 29:182–90. doi: 10.6065/apem.2346090.045 38956754 PMC11220395

[30] Lee J Kang S-C Kwon O Hwang S Moon JS Kim J . Reference values for waist circumference and waist–height ratio in Korean children and adolescents. J Obes Metab Syndrome. (2022) 31:263. doi: 10.7570/jomes22033 36070974 PMC9579477

[31] Ciardullo S Monti T Perseghin G . Prevalence of liver steatosis and fibrosis detected by transient elastography in adolescents in the 2017–2018 National Health and Nutrition Examination Survey. Clin Gastroenterol Hepatol. (2021) 19:384–90:e1. doi: 10.1016/j.cgh.2020.06.048 32623006

[32] Karlas T Petroff D Sasso M Fan J-G Mi Y-Q de Lédinghen V . Individual patient data meta-analysis of controlled attenuation parameter (CAP) technology for assessing steatosis. J Hepatol. (2017) 66:1022–30. doi: 10.1016/j.jhep.2016.12.022 28039099

[33] Zeng J Jin Q Yang J Yang R-X Zhang R-N Zhao J . Prevalence and incidence of MAFLD and associated anthropometric parameters among prepubertal children of the Shanghai Birth Cohort. Hepatol Int. (2023) 17:1416–28. doi: 10.1007/s12072-023-10574-1 37728728

[34] Han SJ Baik SJ Yoon YH Kim JH Lee HS Jeon S . Risk of metabolic syndrome and fatty liver diseases in gastric cancer survivors: a propensity score-matched analysis. Korean J Gastroenterol. (2023) 81:154–62. doi: 10.4166/kjg.2022.113 37096435 PMC12285311

[35] Chiloiro M Caruso MG Cisternino AM Inguaggiato R Reddavide R Bonfiglio C . Ultrasound evaluation and correlates of fatty liver disease: a population study in a Mediterranean area. Metab Syndrome Related Disord. (2013) 11:349–58. doi: 10.1089/met.2012.0169 23758075

[36] Xanthakos SA Ibrahim S Adams K Kohli R Sathya P Sundaram S . AASLD practice statement on the evaluation and management of metabolic dysfunction-associated steatotic liver disease in children. Hepatology. (2025). doi: 10.1097/HEP.0000000000001150 40300071

[37] Friedman SL Neuschwander-Tetri BA Rinella M Sanyal AJ . Mechanisms of NAFLD development and therapeutic strategies. Nat Med. (2018) 24:908–22. doi: 10.1038/s41591-018-0104-9 29967350 PMC6553468

[38] Tilg H Moschen AR . Evolution of inflammation in nonalcoholic fatty liver disease: the multiple parallel hits hypothesis. Hepatology. (2010) 52:1836–46. doi: 10.1002/hep.24001 21038418

[39] Lewis GF Rader DJ . New insights into the regulation of HDL metabolism and reverse cholesterol transport. Circ Res. (2005) 96:1221–32. doi: 10.1161/01.res.0000170946.56981.5c 15976321

[40] Choi Y Yang H Jeon S Cho KW Kim SJ Kim S . Prediction of insulin resistance and elevated liver transaminases using serum uric acid and derived markers in children and adolescents. Eur J Clin Nutr. (2024) 78:864–71. doi: 10.1038/s41430-024-01475-z 39060541

[41] Quijada Z Paoli M Zerpa Y Camacho N Cichetti R Villarroel V . The triglyceride/HDL-cholesterol ratio as a marker of cardiovascular risk in obese children; association with traditional and emergent risk factors. Pediatr Diabetes. (2008) 9:464–71. doi: 10.1111/j.1399-5448.2008.00406.x 18507788

[42] Lee J-H Kim D Kim HJ Lee C-H Yang JI Kim W . Hepatic steatosis index: a simple screening tool reflecting nonalcoholic fatty liver disease. Digestive Liver Dis. (2010) 42:503–8. doi: 10.1016/j.dld.2009.08.002 19766548

