# Supplementary material for: Optimal cutoff values of alanine aminotransferase and its derived markers for pediatric MASLD
Source: Front Endocrinol (Lausanne). 2026 Jul 22;17:1808797. doi: 10.3389/fendo.2026.1808797 (PMC13437452; doi:10.3389/fendo.2026.1808797)
Supplement: Supplementary file 1 [file DataSheet1.pdf]

Supplementary Table 1. Pairwise comparison of AUCs among the parameters for MASLD prediction in NHANES and real-world clinic datasets.

Variables	ALT	ALT/AST	ALT/HDL	TG/HDL	HSI
NHANES					
Total					
ALT	ref	0.003	<0.001	0.494	<0.001
ALT/AST	0.003	ref	0.902	0.011	<0.001
ALT/HDL	<0.001	0.902	ref	0.005	<0.001
TG/HDL	0.494	0.011	0.005	ref	<0.001
HSI	<0.001	<0.001	<0.001	<0.001	ref
Male					
ALT	ref	0.056	<0.001	0.229	<0.001
ALT/AST	0.056	ref	0.695	0.02	<0.001
ALT/HDL	<0.001	0.695	ref	0.004	<0.001
TG/HDL	0.229	0.02	0.004	ref	<0.001
HSI	<0.001	<0.001	<0.001	<0.001	ref
Female					
ALT	ref	0.081	<0.001	0.884	<0.001
ALT/AST	0.081	ref	0.466	0.208	<0.001
ALT/HDL	<0.001	0.466	ref	0.036	<0.001
TG/HDL	0.884	0.208	0.036	ref	<0.001
HSI	<0.001	<0.001	<0.001	<0.001	ref
Real-world clinic data					
Total					

ALT	ref	0.710	0.006	0.053	<0.001
ALT/AST	0.710	ref	0.271	0.034	<0.001
ALT/HDL	0.006	0.271	ref	0.002	0.005
TG/HDL	0.053	0.034	0.002	ref	<0.001
HSI	<0.001	<0.001	<0.001	0.005	ref
Male					
ALT	ref	0.973	0.694	0.037	<0.001
ALT/AST	0.973	ref	0.877	0.04	<0.001
ALT/HDL	0.694	0.877	ref	0.012	<0.001
TG/HDL	0.037	0.04	0.012	ref	<0.001
HSI	<0.001	<0.001	<0.001	<0.001	ref
Female					
ALT	ref	0.473	0.003	0.694	0.063
ALT/AST	0.473	ref	0.134	0.437	0.084
ALT/HDL	0.003	0.134	ref	0.076	0.782
TG/HDL	0.694	0.437	0.076	ref	0.064
HSI	0.063	0.084	0.782	0.064	ref

Note: Delong method was used to perform pairwise comparisons between AUCs for the parameters. Values are presented as *p* values.

Abbreviations: AUC, area under the receiver operating characteristic curve; ALT, alanine aminotransferase; MASLD, metabolic dysfunction-associated steatotic liver disease; NHANES, National Health and Nutrition Examination Survey, AST, aspartate aminotransferase, TG, triglycerides; HDL, high-density lipoprotein cholesterol; HSI, hepatic steatosis index.

Supplementary Table 2. Diagnostic accuracy of the ALT-derived markers for MASLD

Total						
**ALT**						
Cut-off points	%	Sensitivity	Specificity	Accuracy	PPV	NPV
>10	73.58	0.87 (0.83-0.91)	0.31 (0.28-0.34)	0.45 (0.42-0.48)	0.29 (0.26-0.32)	0.88 (0.85-0.92)
>11	62.61	0.85 (0.80-0.89)	0.45 (0.41-0.48)	0.54 (0.51-0.57)	0.33 (0.30-0.37)	0.90 (0.87-0.93)
**>12**	**54.66**	**0.79 (0.74-0.84)**	**0.53 (0.50-0.57)**	**0.59 (0.57-0.62)**	**0.35 (0.32-0.39)**	**0.89 (0.86-0.91)**
>13	47.50	0.74 (0.69-0.79)	0.61 (0.58-0.64)	0.64 (0.62-0.67)	0.38 (0.34-0.42)	0.88 (0.85-0.91)
>14	40.33	0.67 (0.62-0.73)	0.68 (0.65-0.72)	0.68 (0.65-0.71)	0.41 (0.36-0.45)	0.87 (0.84-0.89)
>15	33.33	0.60 (0.54-0.65)	0.75 (0.72-0.78)	0.71 (0.69-0.74)	0.44 (0.39-0.49)	0.85 (0.83-0.88)
>16	29.19	0.54 (0.48-0.60)	0.79 (0.76-0.82)	0.73 (0.70-0.75)	0.46 (0.40-0.51)	0.84 (0.82-0.87)
**>17**	**26.68**	**0.52 (0.46-0.58)**	**0.82 (0.79-0.84)**	**0.74 (0.72-0.77)**	**0.48 (0.42-0.53)**	**0.84 (0.82-0.86)**
>18	23.58	0.47 (0.41-0.53)	0.84 (0.82-0.87)	0.75 (0.73-0.78)	0.49 (0.43-0.55)	0.83 (0.81-0.86)
>19	19.95	0.41 (0.35-0.47)	0.87 (0.85-0.89)	0.76 (0.73-0.78)	0.51 (0.44-0.57)	0.82 (0.80-0.84)
>20	17.70	0.39 (0.33-0.45)	0.89 (0.87-0.91)	0.77 (0.75-0.79)	0.54 (0.47-0.61)	0.82 (0.79-0.84)
**ALT/AST**						
Cut-off points						
>0.0	100.00	1.00 (1.00-1.00)	0.00 (0.00-0.00)	0.25 (0.22-0.27)	0.25 (0.22-0.27)	
>0.1	100.00	1.00 (1.00-1.00)	0.00 (0.00-0.00)	0.25 (0.22-0.27)	0.25 (0.22-0.27)	
>0.2	99.91	1.00 (1.00-1.00)	0.00 (-0.00-0.00)	0.25 (0.22-0.27)	0.25 (0.22-0.27)	1.00 (1.00-1.00)
>0.3	99.14	1.00 (1.00-1.00)	0.01 (0.00-0.02)	0.25 (0.23-0.28)	0.25 (0.22-0.27)	1.00 (1.00-1.00)
>0.4	98.01	0.99 (0.98-1.00)	0.02 (0.01-0.03)	0.26 (0.24-0.29)	0.25 (0.22-0.27)	0.91 (0.80-1.00)
>0.5	89.03	0.96 (0.94-0.99)	0.13 (0.11-0.16)	0.34 (0.31-0.36)	0.27 (0.24-0.29)	0.92 (0.87-0.97)
>0.6	73.58	0.90 (0.87-0.94)	0.32 (0.29-0.35)	0.46 (0.43-0.49)	0.30 (0.27-0.33)	0.91 (0.88-0.94)
**>0.7**	**53.80**	**0.81 (0.77-0.86)**	**0.55 (0.52-0.58)**	**0.62 (0.59-0.64)**	**0.37 (0.33-0.41)**	**0.90 (0.88-0.93)**
>0.8	36.96	0.67 (0.61-0.72)	0.73 (0.70-0.76)	0.71 (0.69-0.74)	0.44 (0.40-0.49)	0.87 (0.85-0.90)
**>0.9**	**27.98**	**0.55 (0.49-0.61)**	**0.81 (0.78-0.83)**	**0.74 (0.72-0.77)**	**0.48 (0.43-0.54)**	**0.85 (0.82-0.87)**
>1.0	18.13	0.42 (0.37-0.48)	0.90 (0.88-0.92)	0.78 (0.76-0.80)	0.57 (0.50-0.64)	0.83 (0.80-0.85)
**ALT/HDL**						
Cut-off points						
>0.0	100.00	1.00 (1.00-1.00)	0.00 (0.00-0.00)	0.25 (0.22-0.27)	0.25 (0.22-0.27)	
>0.1	98.45	1.00 (1.00-1.00)	0.02 (0.01-0.03)	0.26 (0.24-0.29)	0.25 (0.22-0.27)	1.00 (1.00-1.00)
>0.2	70.38	0.87 (0.83-0.91)	0.35 (0.32-0.38)	0.48 (0.45-0.51)	0.30 (0.27-0.34)	0.90 (0.86-0.93)
**>0.3**	**38.00**	**0.68 (0.63-0.73)**	**0.72 (0.69-0.75)**	**0.71 (0.68-0.73)**	**0.44 (0.39-0.49)**	**0.87 (0.85-0.90)**
**>0.4**	**21.85**	**0.50 (0.44-0.56)**	**0.87 (0.85-0.90)**	**0.78 (0.76-0.81)**	**0.56 (0.50-0.62)**	**0.84 (0.82-0.87)**

>0.5	14.16	0.37 (0.32-0.43)	0.93 (0.92-0.95)	0.80 (0.77-0.82)	0.65 (0.57-0.72)	0.82 (0.80-0.84)
>0.6	10.10	0.28 (0.23-0.33)	0.96 (0.94-0.97)	0.79 (0.77-0.81)	0.68 (0.59-0.76)	0.80 (0.78-0.83)
>0.7	7.60	0.22 (0.17-0.27)	0.97 (0.96-0.98)	0.79 (0.76-0.81)	0.72 (0.62-0.81)	0.79 (0.77-0.82)
>0.8	5.87	0.17 (0.13-0.22)	0.98 (0.97-0.99)	0.78 (0.76-0.80)	0.72 (0.61-0.83)	0.78 (0.76-0.81)
>0.9	4.23	0.13 (0.09-0.17)	0.99 (0.98-0.99)	0.77 (0.75-0.80)	0.73 (0.61-0.86)	0.78 (0.75-0.80)
>1.0	3.54	0.12 (0.08-0.15)	0.99 (0.98-1.00)	0.78 (0.75-0.80)	0.80 (0.68-0.93)	0.78 (0.75-0.80)
**Male**						
**ALT**						
Cut-off points						
>10	83.36	0.92 (0.88-0.96)	0.20 (0.16-0.23)	0.39 (0.35-0.43)	0.29 (0.25-0.33)	0.87 (0.81-0.94)
>11	72.92	0.91 (0.87-0.96)	0.34 (0.29-0.38)	0.49 (0.45-0.53)	0.33 (0.29-0.37)	0.92 (0.87-0.96)
>12	65.42	0.87 (0.82-0.92)	0.42 (0.38-0.47)	0.54 (0.50-0.58)	0.35 (0.30-0.40)	0.90 (0.86-0.94)
**>13**	**58.24**	**0.84 (0.78-0.90)**	**0.51 (0.46-0.55)**	**0.60 (0.56-0.63)**	**0.38 (0.33-0.43)**	**0.90 (0.86-0.94)**
>14	50.24	0.79 (0.73-0.85)	0.60 (0.55-0.64)	0.65 (0.61-0.69)	0.41 (0.36-0.47)	0.89 (0.85-0.92)
>15	43.07	0.71 (0.64-0.78)	0.67 (0.63-0.71)	0.68 (0.65-0.72)	0.44 (0.38-0.50)	0.87 (0.83-0.90)
>16	38.99	0.67 (0.60-0.74)	0.71 (0.67-0.75)	0.70 (0.66-0.74)	0.45 (0.39-0.51)	0.86 (0.82-0.89)
>17	36.05	0.66 (0.59-0.73)	0.75 (0.71-0.79)	0.72 (0.69-0.76)	0.48 (0.41-0.55)	0.86 (0.83-0.89)
>18	32.14	0.60 (0.53-0.68)	0.78 (0.74-0.82)	0.73 (0.70-0.77)	0.49 (0.42-0.56)	0.85 (0.81-0.88)
>19	27.90	0.55 (0.47-0.62)	0.82 (0.78-0.85)	0.75 (0.71-0.78)	0.51 (0.44-0.59)	0.83 (0.80-0.87)
**>20**	**24.96**	**0.52 (0.44-0.59)**	**0.85 (0.81-0.88)**	**0.76 (0.72-0.79)**	**0.54 (0.46-0.62)**	**0.83 (0.80-0.86)**
**ALT/AST**						
Cut-off points						
>0.0	100.00	1.00 (1.00-1.00)	0.00 (0.00-0.00)	0.26 (0.23-0.30)	0.26 (0.23-0.30)	
>0.1	100.00	1.00 (1.00-1.00)	0.00 (0.00-0.00)	0.26 (0.23-0.30)	0.26 (0.23-0.30)	
>0.2	100.00	1.00 (1.00-1.00)	0.00 (0.00-0.00)	0.26 (0.23-0.30)	0.26 (0.23-0.30)	
>0.3	99.35	1.00 (1.00-1.00)	0.01 (0.00-0.02)	0.27 (0.23-0.30)	0.26 (0.23-0.30)	1.00 (1.00-1.00)
>0.4	98.04	0.99 (0.97-1.00)	0.02 (0.01-0.04)	0.28 (0.24-0.31)	0.26 (0.23-0.30)	0.83 (0.62-1.00)
>0.5	90.38	0.98 (0.95-1.00)	0.12 (0.09-0.15)	0.35 (0.31-0.38)	0.28 (0.25-0.32)	0.93 (0.87-1.00)
>0.6	76.84	0.94 (0.90-0.98)	0.29 (0.25-0.33)	0.46 (0.42-0.50)	0.32 (0.28-0.36)	0.93 (0.89-0.97)
**>0.7**	**57.42**	**0.83 (0.77-0.89)**	**0.52 (0.47-0.56)**	**0.60 (0.56-0.64)**	**0.38 (0.33-0.43)**	**0.90 (0.86-0.93)**
>0.8	40.95	0.73 (0.66-0.80)	0.70 (0.66-0.75)	0.71 (0.67-0.75)	0.47 (0.40-0.53)	0.88 (0.84-0.91)
>0.9	32.79	0.61 (0.54-0.69)	0.77 (0.74-0.81)	0.73 (0.70-0.77)	0.49 (0.42-0.56)	0.85 (0.81-0.88)
**>1.0**	**22.68**	**0.52 (0.44-0.59)**	**0.88 (0.85-0.91)**	**0.78 (0.75-0.81)**	**0.60 (0.52-0.68)**	**0.84 (0.80-0.87)**
**ALT/HDL**						
Cut-off points						
>0.0	100.00	1.00 (1.00-1.00)	0.00 (0.00-0.00)	0.26 (0.23-0.30)	0.26 (0.23-0.30)	

>0.1	99.67	1.00 (1.00-1.00)	0.00 (-0.00-0.01)	0.27 (0.23-0.30)	0.26 (0.23-0.30)	1.00 (1.00-1.00)
>0.2	81.08	0.92 (0.88-0.96)	0.23 (0.19-0.27)	0.41 (0.37-0.45)	0.30 (0.26-0.34)	0.89 (0.83-0.95)
**>0.3**	**51.06**	**0.83 (0.77-0.88)**	**0.60 (0.56-0.65)**	**0.66 (0.62-0.70)**	**0.42 (0.37-0.48)**	**0.91 (0.87-0.94)**
>0.4	31.00	0.65 (0.57-0.72)	0.81 (0.77-0.85)	0.77 (0.73-0.80)	0.55 (0.48-0.62)	0.87 (0.83-0.90)
**>0.5**	**21.53**	**0.52 (0.44-0.60)**	**0.89 (0.87-0.92)**	**0.80 (0.76-0.83)**	**0.64 (0.55-0.72)**	**0.84 (0.81-0.87)**
>0.6	15.99	0.40 (0.32-0.47)	0.92 (0.90-0.95)	0.79 (0.75-0.82)	0.65 (0.56-0.75)	0.81 (0.78-0.85)
>0.7	12.56	0.33 (0.26-0.40)	0.95 (0.93-0.97)	0.78 (0.75-0.82)	0.69 (0.58-0.79)	0.80 (0.76-0.83)
>0.8	10.28	0.28 (0.21-0.35)	0.96 (0.94-0.98)	0.78 (0.75-0.81)	0.71 (0.60-0.83)	0.79 (0.75-0.82)
>0.9	7.50	0.21 (0.15-0.27)	0.97 (0.96-0.99)	0.77 (0.74-0.81)	0.74 (0.61-0.87)	0.78 (0.74-0.81)
>1.0	6.36	0.20 (0.14-0.26)	0.98 (0.97-1.00)	0.78 (0.75-0.81)	0.82 (0.70-0.94)	0.78 (0.74-0.81)
**Female**						
**ALT**						
Cut-off points						
>10	62.57	0.81 (0.74-0.88)	0.43 (0.38-0.48)	0.52 (0.47-0.56)	0.29 (0.24-0.34)	0.89 (0.84-0.93)
**>11**	**51.01**	**0.76 (0.68-0.83)**	**0.56 (0.51-0.61)**	**0.61 (0.56-0.65)**	**0.33 (0.28-0.39)**	**0.89 (0.85-0.93)**
>12	42.57	0.68 (0.60-0.77)	0.65 (0.60-0.69)	0.66 (0.62-0.70)	0.36 (0.30-0.42)	0.88 (0.84-0.91)
>13	35.41	0.62 (0.53-0.70)	0.72 (0.68-0.77)	0.70 (0.66-0.74)	0.39 (0.32-0.46)	0.87 (0.83-0.90)
**>14**	**29.17**	**0.52 (0.43-0.61)**	**0.77 (0.74-0.81)**	**0.72 (0.68-0.76)**	**0.40 (0.33-0.48)**	**0.85 (0.81-0.88)**
>15	22.39	0.44 (0.35-0.53)	0.84 (0.80-0.87)	0.75 (0.71-0.79)	0.44 (0.35-0.53)	0.84 (0.80-0.87)
>16	18.17	0.37 (0.29-0.46)	0.87 (0.84-0.91)	0.76 (0.73-0.80)	0.46 (0.37-0.56)	0.83 (0.79-0.86)
>17	16.15	0.34 (0.26-0.43)	0.89 (0.86-0.92)	0.77 (0.73-0.80)	0.48 (0.37-0.58)	0.82 (0.79-0.86)
>18	13.94	0.30 (0.22-0.38)	0.91 (0.88-0.94)	0.77 (0.74-0.81)	0.49 (0.37-0.60)	0.82 (0.78-0.85)
>19	11.01	0.24 (0.16-0.31)	0.93 (0.90-0.95)	0.77 (0.74-0.81)	0.48 (0.36-0.61)	0.81 (0.77-0.84)
>20	9.54	0.23 (0.15-0.30)	0.94 (0.92-0.97)	0.78 (0.75-0.82)	0.54 (0.40-0.67)	0.81 (0.77-0.84)
**ALT/AST**						
Cut-off points						
>0.0	100.00	1.00 (1.00-1.00)	0.00 (0.00-0.00)	0.23 (0.19-0.26)	0.23 (0.19-0.26)	
>0.1	100.00	1.00 (1.00-1.00)	0.00 (0.00-0.00)	0.23 (0.19-0.26)	0.23 (0.19-0.26)	
>0.2	99.82	1.00 (1.00-1.00)	0.00 (-0.00-0.01)	0.23 (0.19-0.26)	0.23 (0.19-0.26)	1.00 (1.00-1.00)
>0.3	98.90	1.00 (1.00-1.00)	0.01 (0.00-0.03)	0.24 (0.20-0.27)	0.23 (0.19-0.26)	1.00 (1.00-1.00)
>0.4	97.98	1.00 (1.00-1.00)	0.03 (0.01-0.04)	0.25 (0.21-0.28)	0.23 (0.19-0.27)	1.00 (1.00-1.00)
>0.5	87.52	0.95 (0.91-0.99)	0.15 (0.11-0.18)	0.33 (0.29-0.37)	0.25 (0.21-0.28)	0.91 (0.84-0.98)
>0.6	69.91	0.86 (0.80-0.92)	0.35 (0.30-0.39)	0.46 (0.42-0.51)	0.28 (0.23-0.32)	0.90 (0.85-0.94)
**>0.7**	**49.72**	**0.79 (0.72-0.86)**	**0.59 (0.54-0.63)**	**0.63 (0.59-0.67)**	**0.36 (0.30-0.42)**	**0.91 (0.87-0.94)**
**>0.8**	**32.48**	**0.59 (0.51-0.68)**	**0.75 (0.71-0.79)**	**0.72 (0.68-0.76)**	**0.41 (0.34-0.48)**	**0.86 (0.83-0.90)**
>0.9	22.57	0.46 (0.38-0.55)	0.84 (0.81-0.88)	0.76 (0.72-0.79)	0.46 (0.38-0.55)	0.84 (0.81-0.88)

>1.0	13.03	0.30 (0.22-0.38)	0.92 (0.89-0.95)	0.78 (0.75-0.81)	0.52 (0.40-0.64)	0.82 (0.78-0.85)
**ALT/HDL**						
Cut-off points						
>0.0	100.00	1.00 (1.00-1.00)	0.00 (0.00-0.00)	0.23 (0.19-0.26)	0.23 (0.19-0.26)	
>0.1	97.06	1.00 (1.00-1.00)	0.04 (0.02-0.06)	0.26 (0.22-0.29)	0.23 (0.20-0.27)	1.00 (1.00-1.00)
**>0.2**	**58.35**	**0.81 (0.74-0.88)**	**0.50 (0.46-0.55)**	**0.56 (0.52-0.60)**	**0.31 (0.26-0.37)**	**0.90 (0.86-0.94)**
**>0.3**	**23.30**	**0.50 (0.41-0.59)**	**0.84 (0.81-0.88)**	**0.76 (0.73-0.80)**	**0.47 (0.39-0.56)**	**0.85 (0.81-0.88)**
>0.4	11.56	0.31 (0.23-0.39)	0.94 (0.92-0.96)	0.80 (0.76-0.83)	0.60 (0.48-0.72)	0.82 (0.79-0.86)
>0.5	5.87	0.18 (0.11-0.25)	0.98 (0.96-0.99)	0.80 (0.76-0.83)	0.69 (0.53-0.85)	0.80 (0.77-0.84)
>0.6	3.49	0.12 (0.06-0.18)	0.99 (0.98-1.00)	0.79 (0.76-0.83)	0.79 (0.61-0.97)	0.79 (0.76-0.83)
>0.7	2.02	0.08 (0.03-0.13)	1.00 (0.99-1.00)	0.79 (0.76-0.82)	0.91 (0.74-1.00)	0.79 (0.75-0.82)
>0.8	0.92	0.03 (0.00-0.06)	1.00 (0.99-1.00)	0.78 (0.75-0.81)	0.80 (0.45-1.00)	0.78 (0.74-0.81)
>0.9	0.55	0.02 (-0.01-0.04)	1.00 (0.99-1.00)	0.78 (0.74-0.81)	0.67 (0.13-1.00)	0.78 (0.74-0.81)
>1.0	0.37	0.01 (-0.01-0.02)	1.00 (0.99-1.00)	0.77 (0.74-0.81)	0.50 (-0.19-1.00)	0.78 (0.74-0.81)

Note: The optimal cutoffs for MASLD diagnosis were highlighted in bold

Abbreviations: ALT, alanine aminotransferase; MASLD, metabolic dysfunction-associated steatotic liver disease; PPV, Positive predictive value; NPV, Negative predictive value; AST, aspartate aminotransferase, TG, triglycerides; HDL, high-density lipoprotein cholesterol.

Supplementary Table 3. Cross validation of ALT-derived markers and preexisting composite markers for MASLD in NHANES and real-world clinic data

	Unadjusted model		Adjusted model*	
	AUC (95% CI)	*p* value	AUC (95% CI)	*p* value
NHANES				
Total				
ALT	0.73 (0.66-0.80)	<0.001	0.91 (0.86-0.96)	<0.001
ALT/AST	0.77 (0.69-0.84)	<0.001	0.91 (0.87-0.96)	<0.001
ALT/HDL	0.76 (0.71-0.81)	<0.001	0.91 (0.87-0.96)	<0.001
TG/HDL	0.72 (0.67-0.76)	<0.001	0.91 (0.86-0.96)	<0.001
HSI	0.90 (0.86-0.94)	<0.001	0.91 (0.87-0.96)	<0.001
Male				
ALT	0.75 (0.72-0.78)	<0.001	0.93 (0.90-0.97)	<0.001
ALT/AST	0.78 (0.73-0.84)	<0.001	0.93 (0.90-0.97)	<0.001
ALT/HDL	0.79 (0.75-0.83)	<0.001	0.93 (0.90-0.97)	<0.001
TG/HDL	0.73 (0.63-0.82)	<0.001	0.93 (0.89-0.97)	<0.001
HSI	0.92 (0.88-0.96)	<0.001	0.93 (0.90-0.97)	<0.001
Female				
ALT	0.70 (0.65-0.76)	<0.001	0.89 (0.84-0.94)	<0.001
ALT/AST	0.74 (0.62-0.86)	<0.001	0.89 (0.83-0.95)	<0.001
ALT/HDL	0.75 (0.67-0.83)	<0.001	0.89 (0.85-0.94)	<0.001
TG/HDL	0.70 (0.60-0.80)	<0.001	0.89 (0.86-0.93)	<0.001
HSI	0.89 (0.83-0.95)	<0.001	0.89 (0.84-0.94)	<0.001
Real-world clinic data				
Total				
ALT	0.84 (0.75-0.94)	<0.001	0.94 (0.86-1.01)	<0.001
ALT/AST	0.86 (0.79-0.92)	<0.001	0.94 (0.88-0.99)	<0.001
ALT/HDL	0.87 (0.77-0.96)	<0.001	0.94 (0.87-1.00)	<0.001
TG/HDL	0.76 (0.65-0.87)	<0.001	0.92 (0.88-0.95)	<0.001
HSI	0.92 (0.85-0.99)	<0.001	0.94 (0.89-0.99)	<0.001
Male				
ALT	0.82 (0.73-0.90)	<0.001	0.95 (0.88-1.03)	<0.001
ALT/AST	0.81 (0.72-0.90)	<0.001	0.94 (0.83-1.05)	<0.001
ALT/HDL	0.82 (0.72-0.93)	<0.001	0.95 (0.87-1.03)	<0.001
TG/HDL	0.70 (0.52-0.88)	0.031	0.90 (0.77-1.03)	<0.001
HSI	0.93 (0.87-0.98)	<0.001	0.94 (0.82-1.06)	<0.001

Female				
ALT	0.86 (0.73-1.00)	<0.001	0.95 (0.88-1.02)	<0.001
ALT/AST	0.88 (0.83-0.93)	<0.001	0.94 (0.85-1.03)	<0.001
ALT/HDL	0.92 (0.81-1.02)	<0.001	0.94 (0.85-1.03)	<0.001
TG/HDL	0.84 (0.68-0.99)	<0.001	0.93 (0.88-0.99)	<0.001
HSI	0.92 (0.82-1.02)	<0.001	0.94 (0.86-1.03)	<0.001

Note: Internal validation for diagnostic performance (AUC) was conducted using 5-fold cross-validation.

* Adjusting for age, sex, BMI, TG in the total group and adjusting for age, BMI, TG in male and female group. For the TG/HDL ratio variable, adjustment for TG was omitted in the adjusted model.

Abbreviations: ALT, alanine aminotransferase; MASLD, metabolic dysfunction-associated steatotic liver disease; NHANES, National Health and Nutrition Examination Survey, AUC, area under the receiver operating characteristic curve; CI, Confidence interval; AST, aspartate aminotransferase, HDL, high-density lipoprotein cholesterol; TG, triglycerides; HSI, hepatic steatosis index.
